# Phloroglucinol Mediated Plant Regeneration of *Ornithogalum dubium* as the Sole “Hormone-Like Supplement” in Plant Tissue Culture Long-Term Experiments

**DOI:** 10.3390/plants9080929

**Published:** 2020-07-23

**Authors:** Carloalberto Petti

**Affiliations:** Institute of Technology Carlow, EnviroCORE, DSH, Kilkenny Road, R93 V960 Carlow, Ireland; carloalberto.petti@Itcarlow.ie; Tel.: +353-059-917-5595

**Keywords:** *Ornithogalum dubium*, callus induction, organogenesis, phloroglucinol, regeneration, phytohormones, geophyte

## Abstract

Tissue culture is an essential requirement in plant science to preserve genetic resources and to expand naturally occurring germplasm. A variety of naturally occurring and synthetic hormones are available to induce the processes of dedifferentiation and redifferentiation. Not all plant material is susceptible to tissue culture, and often complex media and hormone requirements are needed to achieve successful plant propagations. The availability of new hormones or chemicals acting as hormones are critical to the expansion of tissue culture potentials. Phloroglucinol has been shown to have certain hormone-like properties in a variety of studies. *Ornithogalum dubium*, an important geophyte species, was used to characterise the potential of phloroglucinol as the sole plant-like hormone in a tissue culture experiment. Tissue culture, plant regeneration, total phenolic and genetic variability were established by applying a variety of methods throughout long-term experiments. Phloroglucinol did induce callus formation and plant regeneration when used as the sole supplement in the media at a rate of 37%, thus demonstrating auxin/cytokines-like properties. Callus formation was of 3 types, friable and cellular, hard and compact, and a mixture of the two. The important finding was that direct somatogenesis did occur albeit more frequently on younger tissue, whereby rates of induction were up to 52%. It is concluded that phloroglucinol acts as a “hormone-like” molecule and can trigger direct embryogenesis without callus formation.

## 1. Introduction

*Ornithogalum dubium*, also known as the Bethlem Star, is an African species belonging to the Hyacinthaceae family [1]. The Hyacinthaceae family has an estimate of 700–900 species, of which 400 species are endemic in Southern Africa, making it one of the predominant and most important geophyte families [1]. The Ornithogalum group is quite diversified and frequently characterised by large and polychromatic flower populations, often coexisting within the same geographical areas. The Ornithogalum group has been reviewed a number of times, and within it, one genus and one subgenus were identified, characterised by a basic chromosome number difference. Ornithogalum at n = 9 is rich in species (circa 250) extending from Africa to the Mediterranean, Saudi Arabia and India. The subgenus Aspasia is characterised by a smaller basal chromosome number, n = 6, and a reduced number of species 12, of which *O. dubium* is included [1]. *Ornithogalum dubium* is an important commercial species for cut flowers and potted plants [2], with an estimated 40 million cut flowers for 2004 [3]. Extensive cross-species hybridization took place to expand the range of naturally occurring flower colours and embryo rescue to recover viable hybrids [3,4,5,6]. Currently, plant biotechnology plays a substantial role in the amelioration of varieties and in the maintenance of clonality. Basic tissue culture and the more advanced bioreactors offer the opportunity for large amounts of production with the maintenance of genetic integrity [7,8]. Essential requirements for tissue culture are phytohormones, allowing for the processes of dedifferentiation and redifferentiation to take place [9]. Plant explants rarely possess high concentrations of endogenous hormones to initiate a process of regeneration and thus the requirements of supplementing the explants with exogenously applied hormones. Essential hormones for the induction of cell growth are auxin IAA (indole-3-acetic acid) and, to a secondary degree, 2,4-D or 2,4-dichlorophenoxyacetic acid, an auxin chemical analogue. Auxins are a class of plant hormones that are involved in a variety of processes including cell growth, division and elongation. Cytokinins are also a class of phytohormones that regulates a variety of developmental processes including shoot formation and root development. The fine-tuning of hormone supplementation is a critical step to optimize the optimal growth process of cells into organised shoots and roots. Somatic totipotent cells can differentiate into adventitious organs through a process known as organogenesis. Caulogenesis is the process of differentiation into shoots which can be followed by rhizogenesis or root formation. The fine balance between auxin and cytokinin often determines the direction of the organogenesis. Broadly speaking, a higher rate of cytokinins to auxins favours caulogenesis whereas the opposite favours rhizogenesis. The initial concentrations of the hormones are equally important to the relative proportions between auxin and cytokinins. The production of somatic embryos to shoots without undergoing a callus stage is known as direct organogenesis. It is known that hormones can, in a complex manner, self- and cross-regulate by means of intermediate interplay, which are transcriptionally fine-tuned [10,11]. This interplay reflects the often-antithetic roles of each hormone’s categories. Auxin and cytokinins are well-established classical hormones used extensively in plant tissue culture and yet a number of unconventional hormones or hormone co-adjuvants have been identified, which are expanding the current network and hormone interplay [12], amongst the most recent, brassinosteroids, terpenoids, stringolactones and basic peptides [13,14,15,16,17]. Furthermore, a number of chemical analogues, such as TDZ (thidiazuron), 2,4-D (2,4-Dichlorophenoxyacetic acid), BTH or benzothiadiazole amongst others, play crucial additive roles in plant tissue culture.

Phloroglucinol is a simple phenolic, 1, 3, 5-benzenetriol characterised by 3 oxidryl groups, and it is the base structure for numerous bioactive molecules resulting from simple or complex biosynthetic pathways and naturally occurring in microorganisms, animals and plants [18]. Phloroglucinols can display an incremental complexity; it is therefore possible to identify monomeric phloroglucinol with varied substitutions, dimeric, trimeric, tetrameric and phlorotannins. Each of these classes can be then further characterised by chemical substitutions, resulting in numerous variants, and for an extensive review, see Singh and Bharate [19]. Phluoroglucinols, from their simplest form to the more complex structures, have demonstrated an extreme variety of bioactive properties including antimicrobial properties such as antiviral, antibacterial and antifungal properties [20,21,22,23,24,25,26]; antidepressant effects [27,28]; human keratinocyte proliferation ability [29]; antioxidant and cytotoxic activities [30,31]; and anticancer [32,33] and neuroprotective effects [34]. This extreme variation in structures and related functions suggest a pleomorphic functionality of the basic phloroglucinol which is supported by a near ubiquitous presence in the plant kingdom. The ecological and functional roles of phloroglucinols in plants are not well characterised. Phloroglucinol basic structured is modified though a variety of diverse secondary metabolite pathways in a large complexity of molecules which have been extensively reviewed in [18], to which the reader is referred for further information.

As phloroglucinol is a basic phenolic, it has been postulated to have a role in the cell wall and therefore also a potential one in tissue culture, in particular, with regard to the phenomenon of hyperhydricity or vitrification. In fact, in a number of studies, phloroglucinol used in association with other hormones has been shown to enhance root induction, formation and elongation [35,36,37,38,39] along with reducing plant hyperhydricity [40,41,42]. However, the role(s) of phloroglucinol in plant tissue cultures is still not fully understood. It is currently postulated that phloroglucinol functions as an auxin synergist or protectant [43], which would be in agreement with the predominant role observed in root stimulation, formation and, partially, shoot induction [44,45].

In this study, the role of phloroglucinol as the sole “hormone-like supplement” in tissue culture was explored using *O. dubium* as the target species and was compared to standard auxins (IAA)/cytokinins (TDZ and benzylaminopurine (BAP)). Conclusions are drawn on the potential of *O. dubium* to be used as a model geophyte organism.

## 2. Results

### 2.1. Media Assessment and Hormones Baseline Establishment

The propensity of tissue culture of *O. dubium* regeneration was established on 2 types of media, MS (Murashige and Skoog *medium)* basal medium and MS with Gamborgs vitamins (MSG), and with the same hormones and hormone concentrations. Both media formulations displayed an efficient regeneration propensity, with most fragments being able to dedifferentiate to a calli-status and to redifferentiate to a shoot status, without any requirements to alter the media composition. While both media exerted similar effects on the ability of *O. dubium* fragments in organogenesis, the MS media was slightly more effective in such a process (*p* < 0.05), which was then utilised as the preferential medium for the assessment of phloroglucinol.

**Figure 1:**
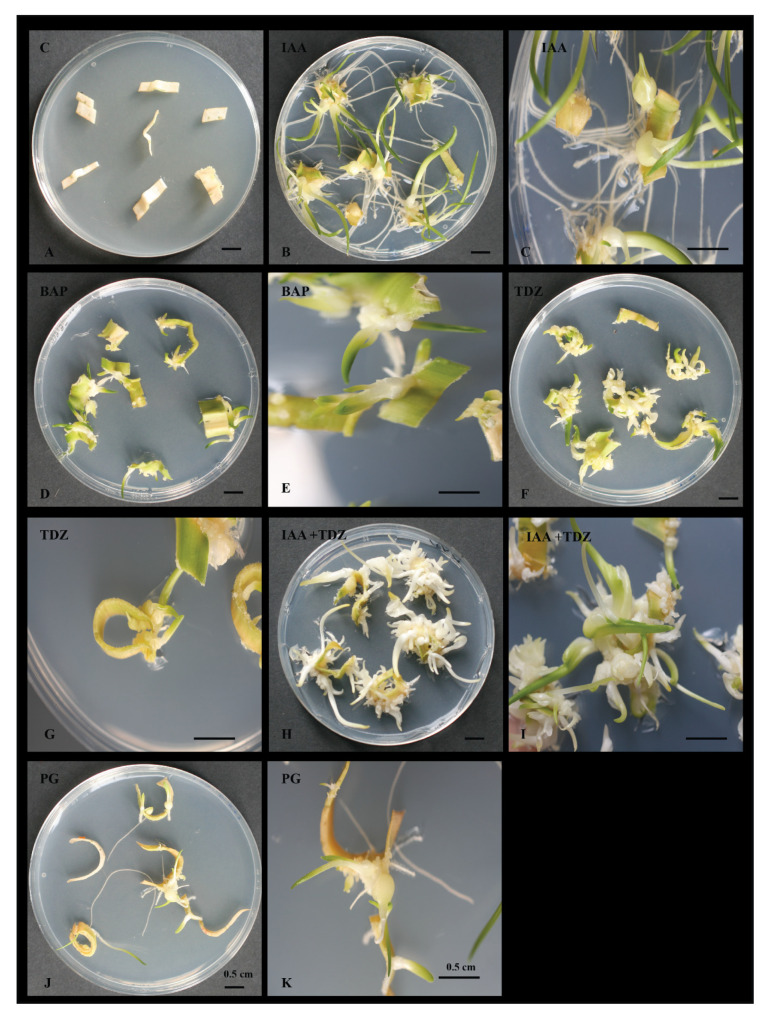
Regeneration properties of different hormones on *Ornithogalum dubium* explants: In (**A**), control fragments show complete bleaching. In (**B**,**C**), the effects of indole-3-acetic acid (IAA) are predominantly seen in extensive root formation. In (**D**,**E**), poorly organised clamps of calli are a response to benzylaminopurine (BAP). In (**F**,**G**), larger clamps and microbulb formation characterise the effect of TDZ supplementation. In (**H**,**I**), larger clamps with well-developed shoots respond to the combination of IAA + TDZ. In (**J**,**K**), shoots, microbulbs and roots are seen as a response to, PG (Phloroglucinol) application. All hormones were at the same concentration (1.5 mg/L). PG concentration was 4 mg/L.

All hormones (IAA, TDZ, BAP and IAA + TDZ) and tested concentrations (0.5, 1.0 and 1.5 mg/L) were able to induce cellular proliferation and ultimately shoot/root formation (Figure 1 and Table 1). As a whole, the rate of efficacy of callus/shoot formation ranged between 52% and 96%. The highest rate was generally obtained with the highest concentration of the supplied hormone (1.5 mg/L), and that was independent of the hormone type that was utilised (Table 1). No callus or shoot formation was observed in the control experiment, whereby no hormones where used in the media (Figure 1 and Table 1). Despite the rates of induction being similar, some noticeable morphological differences could be seen amongst the hormones. In general, BAP (Figure 1C) induced leafy and poorly organised callus/shoots while TDZ appeared to produce preferentially microbulbs (Figure 1D) and IAA increased more callus/leafy shoots and root prone (Figure 1B). The combination of IAA and TDZ (Figure 1E) appeared to increase the rate of calli/shoots formation by up to 20% (min 8%, max 20%) and supported a more optimal morphological development than the independent hormones. However, of the three concentrations utilised, a more effective morphological development took place at the intermediate concentration (1.0 mg/L) and this was the general case for all hormones, although the rate of increment was more noticeable when IAA and TDZ were used together (Table 1).

### 2.2. Phloroglucinol as an Auxin-Like Hormone

**Figure 2:**
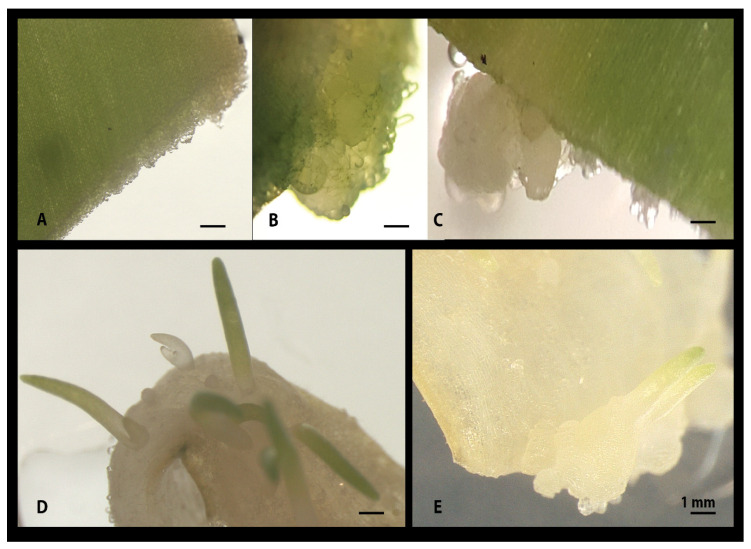
Phloroglucinol mediated organogenesis on *O. dubium* explants. Panel (**A**) through panel (**C**) show callus types regenerated from *O. dubium* fragments: (**A**) friable cellular callus type, hard and compacts (**B**), and a mixture of the 2 types (**C**). In panel (**D**), evidence of multiple direct somatogenesis shoots formation without callus induction is shown. In panel (**E**), microbulbs and shooting primordia are shown.

Phloroglucinol (PG) acted as a hormone by means of stimulating callus induction and organogenesis in shoots and/or microbulbs and roots (Figure 1F and Figure 2). The range of concentrations used in this investigation varied from 1 to 10 mg/L, and at each concentration, phloroglucinol did induce somatogenesis at different rates. Overall, the induction rates varied between 25 and 37% (Table 2). No callus or shoot formation was observed in the control experiment without PG (Figure 1). The most effective concentration was found to be 4 mg/L (Table 2) with an induction rate of 37.5%. Time required for organogenesis induction was found to be a variable characteristic of the phloroglucinol modus operandi, with lag periods observed often between 2 and 4 weeks before visible calli/shoots could be noted. However, variability within these observations were frequent. Such a variability was also a common feature of the fragments’ response to PG, whether none or all fragments within an experiment could regenerate. The callus, when formed, was mainly of three types: cellular and unorganised, hard and microbulb-structured, and a mixture of the 2 types above (Figure 2). Independently from the type of callus formed, if formed later, cellular reorganization would take place, leading to somatogenesis, with well-developed vegetative shoots, single or multiple microbulbs developing vegetative shoots, and root formation. In fewer cases, root formation was initiated before calli and/or shoots.

### 2.3. Phloroglucinol and Callus Induction: Young versus Older Tissue Type

Preliminary evidence suggested that phloroglucinol was more effective on callus/shoot induction on younger tissue than older ones. To support this observation, sampling material from sterile regenerated plantlets were used along with older glasshouse grown plants. In general, there was a statistically significant increase in the rate of callus/shoot induction when younger tissues were compared to older tissue (*p* < 0.05). An increased variability was however observed when it came to identifying the most effective concentration of phloroglucinol. All concentrations had a positive effect with a variable percent of calli/shoots formed for any of the concentrations tested (Table 3). However, rather than a specific optimal concentration, a range of concentrations was determined as optimal, these being between 1 and 4 mg/L. The variability observed was at the plate level as well as at the concentration level. Within any given experiment and any given concentration, the whole set of fragments in a plate (n = 8–10) could be forming calli/shoots or all could be bleaching out and dying. No callus or shoot formation was observed in the control experiment containing no phloroglucinol.

### 2.4. Plant Transplant Vigour and Phloroglucinol

**Figure 3:**
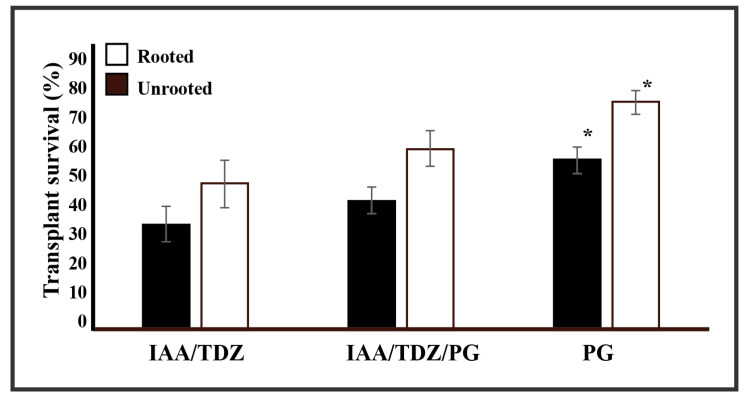
Phloroglucinol-mediated transplant survival rates: the image shows the comparison of survival rates of three hormone treatments derived from rooted and unrooted plant material. * indicates significance at the α value of 0.05.

Tissue culture-derived plantlets require controlled conditions to enable the transition between a sterile environment, the medium, to a non-sterile and complex medium, the compost. The alteration of the growth conditions can dramatically influence the ability of the rooted plantlets to survive the transplant. Anecdotal observations supported phloroglucinol-derived plantlet resistance to the transplant process. To validate such observations, 3 independent sets of experiments over a 12-month period were established and compared the ability of tissue culture rooted and unrooted to survive the transplant process following 3 treatments (Figure 3). In general, there was no difference between the use of sterilised compost versus non-sterilised compost (data not shown). In fact, the impact that sterilised compost had onto the ability of the explants to survive the transplant process was often more dramatic. Based on this evidence, the use of non-sterile compost was preferred. In no case was 100% survival rate attained independent of the treatments considered. However, the plantlets resulting from the phloroglucinol-only treatment were able to survive at a significantly greater rate (78%, *p* < 0.05) than those of the auxin/cytokinin treatment (50%) and the amended phloroglucinol auxin/cytokinin treatment (62%). This general trend was also observed for plant material (microbulbs or multiple shoots) that was not rooted. These showed a marginal increment in the observed survival rates, which were also found to be significant (*p* < 0.05).

As phloroglucinol is a simple phenolic compound, we postulated that the cell wall characteristic of the 3 treatments, IAA + TDZ, IAA + TDZ + PG, and PG, could be altered. Phenotypic characterization of the cell wall of selected lines did not show any obvious difference (data not shown); however, total phenolics were 3.5-fold higher in the phloroglucinol-only resulting plantlets, whereas the amended auxin/cytokinin was 1-fold higher than the unamended treatment (Figure S2). This result was true for the plant material developing on the regeneration media and on younger potted plants; however, on older potted plants, no further difference was found (*p* > 0.05).

### 2.5. Morphological Characteristics

Plantlets regenerated from media supplemented with either IAA, PG, TDZ, BAP or hormone combinations did not differ from each other at a macroscopic level. However, a number of mutants or variants arose from independent experiments (Figure S1). The frequencies of variants were, across the whole set of replications and experiments, in the range of 0.01–0.8%. The main morphological changes were leaf variegation (0.4%, Figure S3), leaf curling (0.08%) and flower morphology (Figure S4). The flower morphology alterations were the most notable and included “double flower or flore pleno”, with partial or total duplication or triplication of tepal number (6 to 12, 15 or 18), acarpous or lack of pistil, and astemonous partial or total lack of stamens (Figure S4). Normal flower morphology was the dominant type occurring, but the occasional altered flower was often seen in a spike of normally developed ones and this/these aberrant flower(s) was/were often the terminal or subterminal ones. The genetic analyses showed substantial similarities amongst the investigated variants. However, a greater degree of variability in terms of frequency of band formation was observed in 2 variants, namely leaf variegated and leaf curling (Figure S5). These data support the evidence of occurrence of some somaclonal variation in tissue culture and, thus, the potential for the attainment of new breeding stocks/varieties.

**Figure 4:**
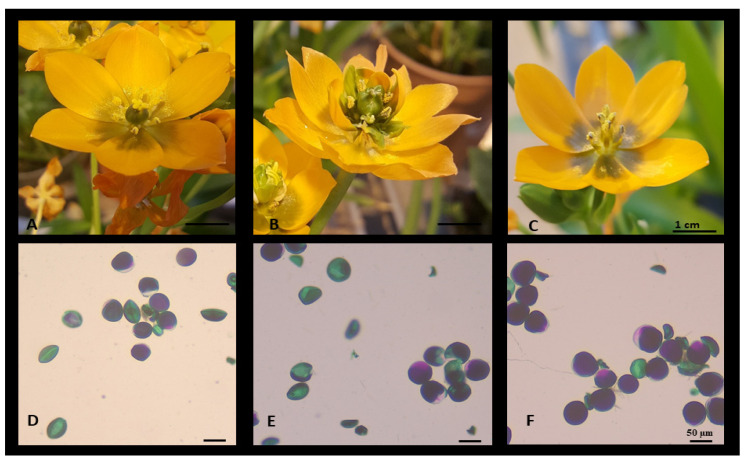
Pollen morphology of 2 selected variants compared to a control line: Panels (**A**) and (**D**) show a normal flower and its pollen phenotype, panels (**B**) and (**E**) show a variant *flore pleno* with altered tepals number and relative pollen, and panels (**C**) and (**F**) show an acarpous flower type and related pollen. The purple colour indicates viable pollen grains, whereas green is an indication of nonviable pollen grains.

Overall, sexual incompatibility was seen to be the predominant condition for self- and cross-pollination experiments. There was no difference whether the pollen used was from a male-only flower, a normal flower or a flore pleno flower (*p* > 0.05). The analyses of pollen viability [46] (Figure 4) showed that all flowers had a degree of pollen grains being fertile and viable; this percentage decreased as the flower morphology alteration increased with the flore pleno type having the least viable pollen grains (*p* < 0.05, Figure 4, 55%), the normal flower had a 65% viability and the acarpous showed the highest at 70%. However, no pollen was found to be entirely nonviable in any given type of flower morphs. Notably, the greater degree of aborted pollen grains was in the acarpous type, which made up the majority of nonviable grains compared to the normal and flore pleno types. No obvious alterations were noted at the tubers level, with microbulbs to fully developed bulbs no different from each other (Figure S5).

## 3. Discussion

Tissue culture in plant science fulfils a variety of roles, from multiplication to maintenance of genetic purity, expansion of germplasm’s diversity and preservation of plant genetic resources. This last one is becoming more and more important with the ever-increasing number of threatened plant species. In a 2002 report by Pitman and Jorgensen [47], the range of threatened species fell between 22 and 47%, while a more recent report by Kew’s Botanical Garden sees this estimate in the order of 22%. No matter which rate is considered, a concerningly large amount of the world’s plant diversity is at risk of extinction.

An optimal balanced and efficacious plant tissue culture protocol is required to enable plant propagation. The ideal media is one that has fewer hormones and that will suffice in its requirements to induce cellular de- and redifferentiation without any additional manipulations. In this report, a highly efficient single medium system was provided with the combination of IAA and TDZ, whereby virtually 100% of the explants de- and redifferentiated on the same medium to form plantlets with roots. However, previous reports also demonstrated the suitability of *O. dubium* in tissue culture; in general, multiple media types were required to obtain calli, shoots and roots [48,49,50,51,52]. A reference background protocol by Watad et al., 1998 [53] was also used in other studies which addressed transformability, for instance [49,50,51,53,54,55]. The protocols used an induction media, followed by a regeneration media which used BA (6-Benzylaminopurine) and NAA (1-Naphthaleneacetic acid) as a combination of a synthetic cytokinin and auxin, which is in fact similar to our protocol with an auxin/cytokinin effect provided by TDZ and a classical auxin, IAA. Importantly though was also the finding that a single hormone, IAA, TDZ or BAP, was equally able to induce callus and microbulb formation and ultimately shoot regeneration (Figure 1 and Table 1), albeit their efficacy was lower than the combination TDZ/IAA. These findings highlight a great sensitivity and response of *Ornithogalum dubium* to hormone manipulations, which could support the species as a model tool for geophyte studies.

Notwithstanding the results above, the intention of the study was to establish the tissue culture abilities of PG, which has not been previously studied on *Ornithogalum dubium*, not alone as the sole hormone or in combination with other hormones. PG unquestionably demonstrated the ability to induce both cellular and friable, and hard and embryogenic callus (Figure 1 and Figure 2) as well as the ability to induce direct somatogenesis or somatic embryogenesis (Figure 2D), however, at rates significantly lower than the comparative auxin/cytokinin protocol (*p* < 0.05). Moreover, the cellular response of *O. dubium* fragments to phloroglucinol was longer and slower than that of TDZ/IAA. Notably, in this study, a trend was observed in which younger tissues were more likely to display somatic embryogenesis (that is intended to be callus free) than the older tissues; thus it is tempting to speculate on the potential link existing between the difference in cell wall reinforcements between the 2 tissue types and the direct somatogenetic responses. Somatic embryogenesis implies a sophisticated chromatin regulation and response to internal and external cues [56,57,58]. It is a powerful ability of plant tissues and plant biotechnology allowing for the recovery of single cell mutants. Somatic embryogenesis was shown to be characterised by a gross similarity to zygotic embryogenesis, including the activation of the same genes and asymmetric cellular division and auxin gradient establishment [56,57]. In arabidopsis, a series of elegant studies demonstrated that there are at least 2 pathways to SE, the first, callus-free and *LEC1* dependent [59], and the second through the *WUS*-dependent pathway [60,61,62]. Interestingly, a number of cell wall-modifying enzymes are also expressed during the process, such as xyloglucan endotransglycosylases [63], class IV endochitinase or extracellular protein 3 [64], nonspecific lipid transfer proteins [65] and arabinogalactan proteins, amongst many others [56]. It is tempting to postulate the existence of a possible link between the simple phenolic structure of PG and its potential role in cell wall and somatic embryogenesis as multifarious and is in need to be further characterised. Phloroglucinol, at least in brown algae, was demonstrated to be the basic unit involved in the biosynthesis of phlorotannins [66,67] and polymerised in various phlorotannins’ structures, which in their insoluble forms are cross-linked to the cell wall [68]. Phenolic crosslinking and reinforcements are natural responses of the plant cell wall to a number of phenomena including pathogen responses, water and other environmental stresses [69,70,71].

*Ornithogalum dubium* total phenolic profile alteration is a noticeable finding; while it seems to be likely connected with the PG potential role in cell wall function, alternative scenarios could exist. For instance, TDZ was able to induce somatic embryogenesis in tissue culture of blueberries with a persistent alteration of the phenolic profiles [72]. The authors suggested that the alteration of the phenolic profiles is due to a direct effect of TDZ on the pathway to phenolics, the shikimate pathway, and this seems to reinforce the potential “hormonal” role of PG. Chemical analogues of natural hormones have been shown to induce an increase in somatic differentiation, organogenesis and rooting on *O. dubium* and *O. thyrsoides* [52]. Calli induction was obtained by standard protocol using NAA and BAP, which were then exposed to BTH with a substantial increase in shoot formation. Elsewhere, shoot induction was observed as a result of medium supplemented with PG at a rate similar to other tested hormones, although the rate did increase by 25% when PG was used in combination to other hormones [73].

*Ornithogalum dubium* is characterised by a significant sensitivity to viral and bacterial infections that are limiting factors inherently bound to vegetative propagation. The susceptibility of *O. dubium* to soft rot is a major curtaining factor to its more widespread cultivation. A qualitative approach combined GFP (Green Fluorescent Protein) and flow cytometry to identify suitably tolerant/resistant cultivars [74]. Genetic engineering was also used to produce plants that were resistant to viral infection [50] and to *Erwinia carotovorum* [51]. In both cases, multiple media were required to ensure the recovery of transgenic plants. The occurrence of somaclonal variation in tissue culture, as shown in this report, is not unknown and has been used as a major source of germplasm variability [75,76], which could be capitalised on to screen and identify new resistant sources as well as phenotypic variants (Figures S3 and S4). A single variant can become the progenitor to many through tissue culture, thus providing a scope for agricultural improvement. The irregularity by which *O. dubium* explants responded to PG suggest that, rather than possessing a hormone-like action, it might possess more likely a hormone helper/developer action. However, since the control experiments did not show any callus/shoot formation, a degree of organogenetic properties must be connected with PG. The likely fine-tuning of PG required for the sole application in plant tissue culture entails more molecular studies; importantly are the unanswered questions of whether PG protects auxin as suggested or even contributes to auxin production? Does PG response depend on the plant genotype? As often observed for other hormones [77] and ultimately, can the ability of direct somatogenesis be harnessed in a more controlled manner for more specialised plant biotechnological applications?

Relevantly so it is the ability and suitability of *O. dubium* to respond to a variety or hormones and to be transformable, the ease of recovering plant material makes it a perfect candidate to become a developing model for geophytes genetic studies.

## 4. Materials and Methods

### 4.1. Original and Produced Plant Material

*Ornithogalum dubium* was acquired commercially from local nurseries; plants were produced in Holland and imported for resale in Ireland through international supply chains. Plants were kept in a temperature-controlled glasshouse at the constant temperature of 22 °C (±2 °C) and a photoperiod of 16 h daylight and 8 h darkness. Plant materials developed subsequently to tissue culture were kept in the same conditions, were repotted in a mixed compost (John Inns 3), and were fertilised and watered according to requirements.

### 4.2. Tissue Culture

Leaf samples from the original plant material were sterilised under standard conditions: ten minutes of sodium hypochlorite (1% active principle) followed by a rinse in sterile distilled water. The material was then exposed to a 10 min wash in 70% ethanol and a repeated number of rinses in sterile water. The plant material was then axenically cut in fragments of circa 0.5 cm in diameter and 1–2 cm in length.

The basic regeneration medium was composed of MS basal salt full strength or MS with Gamborg vitamins (MSG), 3% sucrose; solidified with 0.9% phytoagar; and sterilised under standard conditions (121 °C, 15 PSI, 15 min). Hormones (Indole acetic Acid (IAA); Thidiazuron (TDZ), Kinetin (Kin) and Benzylaminopurine (BAP) were added, alone or in combinations, to the media in axenic conditions to the final concentrations of 0.5, 1 and 1.5 mg/L, respectively. Previous published studies demonstrated a great range of concentrations of PG utilised from uM to mM [43]; based on these, an initial arbitrary choice of 0.5 and 1.0 mg/L was used for pretesting experiments. Phloroglucinol was used alone or in combination with hormones to the final concentration of 1, 2, 4, 10 and 20 mg/L. All media and hormones were obtained from Duchefa (Haarlem, The Netherlands). Stock hormones were prepared in water, DMSO (Dimethyl Sulfoxide) or ethanol according to specific hormone requirements and kept at −20 °C. Hormones were added to the media in sterile conditions and at a media temperature of 50 °C.

Sterile explants (n = 8–10 fragments/plate) were then placed onto the regeneration media supplemented with a single hormone or combinations of hormones, and the parafilm-sealed plates were placed in an incubator (no light) at a constant temperature of 22 °C (±2 °C). Experiments were repeated a minimum of 5 times over a 2-year assessment with a total of 250 fragments exposed onto each medium and hormone or hormone combination types. Fragments were monitored for callus/shoot/microbulb development and regularly subbed on a new medium of the same composition type every 3/4 weeks. Well-developed plantlets/microbulbs were transferred to a growth chamber (Conviron, Isleham, UK) with a photoperiod of 16/8 h daylight/night at a constant temperature of 22 °C (±2 °C). Upon chlorophyll development and root formation, plantlets, solitary or in multiple foci/groups, were either planted in compost and transferred to the glasshouse in the same light and temperature conditions as described above or used as a primary material for a new callus/shoot-induction cycle.

### 4.3. Phenotypical Characterization

Examination of the plant material regenerated over a 2-year period was completed for leaf morphology, flower formation, flower size and bulb size. Morphological variants were isolated and further characterised at DNA levels. Cell wall morphology of selected candidates was evaluated using Calcofluor white (Sigma, Dublin, IE) and UV-fluorescence microscopy. Pollen viability was established by a modified Alexander stain protocol [46] using 10 independent counts of 100 pollen grains from selected variants. For total phenolics analyses, the Folin Ciocalteu methods were used. In brief, 100 mg of fresh plant material was extracted in 80% methanol after crushing it with a mortar and pestle, followed by a heating step (50 °C) for 30 min and 1 mL of the extraction used in the reaction. Absorbance was read at 750 nm. The analyses were completed in triplicate (technical and biological reps). Total Phenolics were expressed as gallic acid (Sigma, UK) equivalents.

### 4.4. DNA Extraction and RAPD Analysis

DNA extraction was completed by using the CTAB (Cetrimonium bromide) method. DNA was quality verified by gel electrophoresis and quantified by nano-drop (Denovix, Wilmington, DE, USA) estimation. Prior to the RAPD (rapid amplified polymorphic DNA) analyses, the DNA was standardized to a similar concentration (20 ng/uL). Twenty RAPD primers (Table S1), 10 bases long, were used in combination to obtain a map of the genetic variability within selected clones from the original single clonal source. Standard PCR reagents and reaction conditions were used with a basic annealing temperature of 45 °C and an extension time of 2.0 min. PCR products were visualised and scored for band presence and absence on a 3% high separation Agarose gel (Sigma, UK). Band pattern was examined using GenAlEx 6.5 [78].

### 4.5. Statistical Analyses

All analyses were performed on SPSS v. 25 (IBM). For nonparametric count data, Chi-square (simple or crosstab) test was used. For parametric continuous data. ANOVA was used followed by post hoc Tukey’s test. Homogeneity of variance was tested by Levene’s Test and Kolmogorov–Smirnov test for normality. Significance was established at the alpha value of 0.05.

## 5. Conclusions

In this report, evidence is provided for an optimised tissue culture protocol for *O. dubium*, which requires no further manipulation to go from plant explants to plantlets. In addition, the response of *O. dubium* to PG is detailed. While the rates of regenerant are significantly lower than the TDZ/IAA protocol, it provided evidence of direct somatogenesis not via callus induction, which can be capitalised upon for the recovery of single cell transformant/mutants. A number of naturally occurring variants were also characterised as proof of concept. Tolerance to transplant shock was also reported to be enhanced by the application of PG. It is suggested that *O. dubium* can become a model for geophytes genetic studies due to its remarkable response to a variety of hormones, the ease of tissue cultures, the potential for direct somatogenesis and ultimately the ease of transformation

## Figures and Tables

**Table 1:** Effects of medium and hormone types on the efficacy of callus/shoot induction in *Ornithogalum dubium.*

		MS Basal Salts		MS Plus Gamborg Vitamins	
Hormone	Concentration (mg/L)	Callus/Shoot Formation (%)	^1^ Average Callus/Shoot Size (cm) ± SD	Callus/Shoot Formation (%)	^1,2^ Average Callus/Shoot Size (cm) ± SD
None	N/A	0	0	0	0
**IAA**	0.5	65.9 ^a^	0.88 ± 0.24 ^a^	64.6 ^a^	0.97 ± 0.17 ^a^
	1	77 ^b^	1.31± 0.34 ^bc^	74.1 ^b^	1.19 ± 0.25 ^b^
	1.5	75.7 ^b^	1.14 ± 0.14 ^b^	74.3 ^b^	0.82 ± 0.33 ^a^
**TDZ**	0.5	61.1 ^a^	0.92 ± 0.25 ^a^	55.9 ^a^	0.98 ± 0.25 ^a^
	1	75.6 ^b^	1.50 ± 0.33 ^bcd^	71.1 ^b^	1.18 ± 0.41 ^b^
	1.5	78.7 ^bc^	1.01 ± 0.45 ^a^	77.2 ^bc^	0.95 ± 0.36 ^a^
**IAA + TDZ**	0.5	82.1 ^b^	0.89 ± 0.25 ^a^	77 ^b^	0.98 ± 0.25 ^a^
	1	90.6 ^bc^	1.63 ± 0.36 ^bce^	81 ^b^	1.62 ± 0.35 ^bc^
	1.5	92 ^bcd^	1.32 ± 0.17 ^bc^	86 ^bc^	1.34 ± 0.27 ^b^
**BAP**	0.5	52.4 ^a^	0.76 ± 0.22 ^a^	55 ^a^	0.72 ± 0.31 ^a^
	1	65 ^a^	1.28 ± 0.15 ^bc^	69.9 ^a^	1.33 ± 0.32 ^b^
	1.5	71.6 ^b^	1.09 ± 0.35 ^b^	72.9 ^b^	1.25 ± 0.45 ^b^

^1^ The average size was established after 4–6 weeks of callus/shoot induction. ^2^ Different letters indicate significant difference (*p* < 0.05).

**Table 2:** Phloroglucinol effects on tissue culture of *Ornithogalum dubium.*

Phloroglucinol Concentration (mg/L)	Callus/Shoot Formation (%)	^1,2^ Average Callus/Shoot Size (cm) ± SD
0	0	0
1	25.2 ^a^	0.83 ± 0.36 ^a^
2	33.3 ^b^	0.87 ± 0.52 ^a^
4	37.5 ^bc^	1.51 ± 0.47 ^bc^
10	32.1 ^b^	1.12 ± 0.49 ^b^
20	27.8 ^a^	1.08 ± 0.53 ^a^

^1^ The average size established after 4–6 weeks of callus induction. ^2^ Different letters indicate significant difference (*p* < 0.05).

**Table 3:** Phloroglucinol effects on tissue culture of young *Ornithogalum dubium* tissues.

Phloroglucinol Concentration (mg/L)	Callus/Shoot Formation (%)	^1,2^ Average Callus/Shoot Size (cm) ± SD
0	0	0
1	37.2 ^a^	0.90 ± 0.25 ^a^
2	44.5 ^b^	1.12 ± 0.36 ^b^
4	52.1 ^bc^	1.65 ± 0.51 ^bc^
10	37.8 ^a^	1.08 ± 0.44 ^a^
20	33.1 ^a^	1.12 ± 0.21 ^a^

^1^ The average size established after 4–6 weeks of callus induction. ^2^ Different letters indicate significant difference.

## Supplementary Materials

The following are available online at https://www.mdpi.com/2223-7747/9/8/929/s1, Figure S1. Morphological characterisation of different clumps of developing plantlets of *O. dubium.* Figure S2. In panel (A), standard curve was generated using Gallic Acid (GA) as a monomeric phenolic. Figure S3. Morphological characteristic of the *O. dubium* leaf variegation variant. Figure S4. Plant phenotyping of selected *Ornithogalum dubium* lines showing various morphological alteration of the flower development of *O. dubium*. Figure S5. In (A) is an example of a polymorphic primer (S24) applied to 7 variants. Table S1. RAPD primers sequences used in the genetic characterization of selected variants.
