# Supplementary material for: Phloroglucinol Mediated Plant Regeneration of Ornithogalum dubium as the Sole “Hormone-Like Supplement” in Plant Tissue Culture Long-Term Experiments
Source: Plants (Basel). 2020 Jul 23;9(8):929. doi: 10.3390/plants9080929 (PMC7464755; doi:10.3390/plants9080929)
Supplement: Supplementary file 1 [file plants-09-00929-s001.pdf]

Supplementary materials

This document contains: Supplementary Table 1 describing the primers used for the genetic characterisation of selected lines and 5 supplementary figures.

Table S1. RAPD primers sequences used in the genetic characterization of selected variants.

Primer Name	Primer sequence (bp)
S21	CAGGCCCTTC
S22	TGCCGAGCTG
S23	AGTCAGCCAC
S24	AATCAGCCAC
S25	AGGGGTCTTG
S26	GGTCCCTGAC
S27	GAAACGGGTG
S28	GTGACGTAGG
S29	GGGTAACGCC
S30	GTGATCGCAG
S31	CAATCGCCGT
S32	TCGGCGATAG
S33	CAGCACCCAC
S34	TCTGTGCTGG
S35	TTCCGAACCC
S36	AGCCAGCGAA
S37	GACCGCTGTT
S38	CAAACGTCGG
S39	AGGTGACCGT
S40	GTTGCGATCC

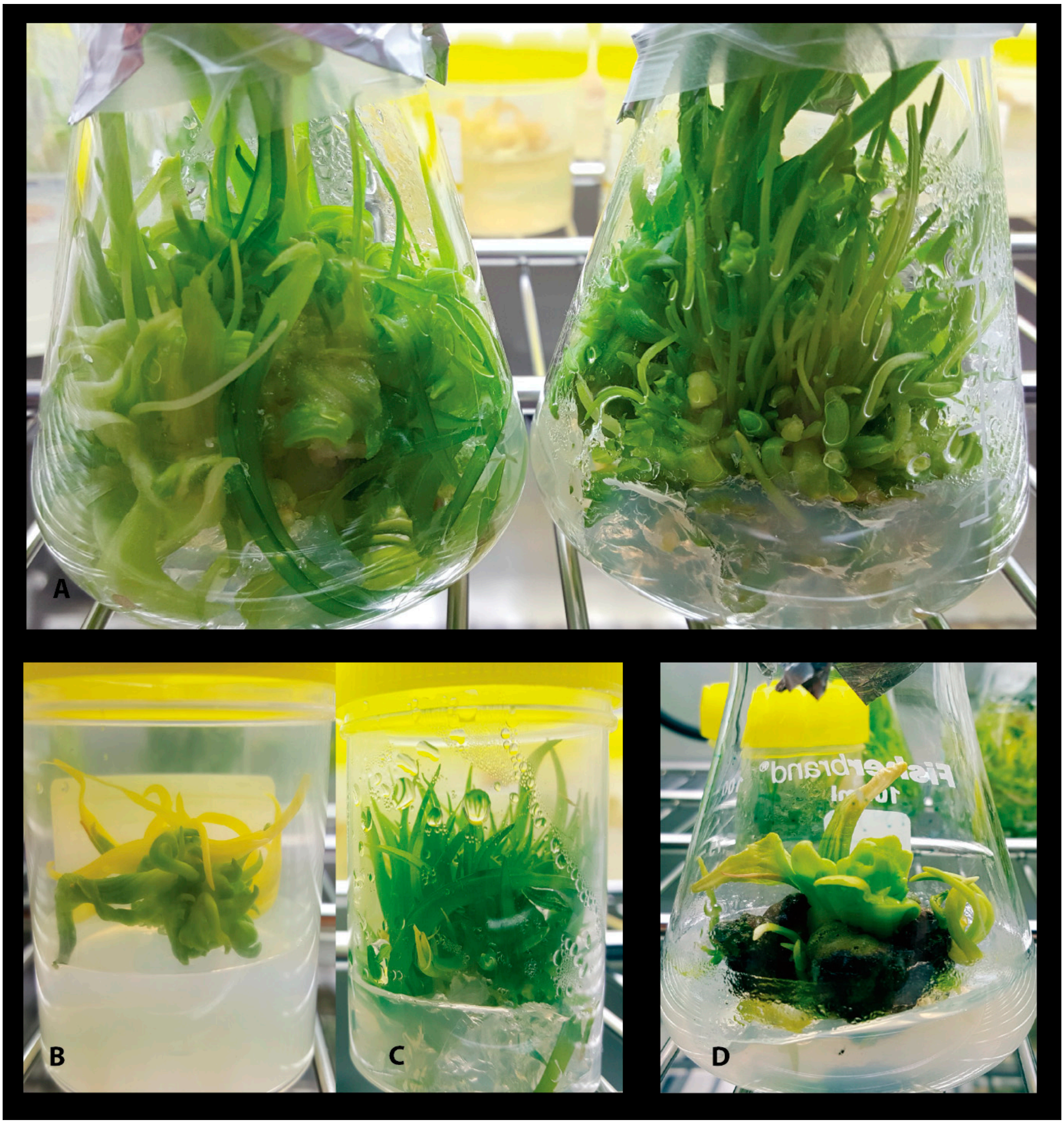

Figure S1. Morphological characterisation of different clumps of developing plantlets of *O. dubium*. In A, IAA+TDZ (1.0/1.0 mg/l). In B IAA+TDZ plus PG2. In C IAA+TDZ and Kin and in D shoot formation of *Hyacinthus orientalis* under similar conditions and used as an outsider in the tissue culture experiments.

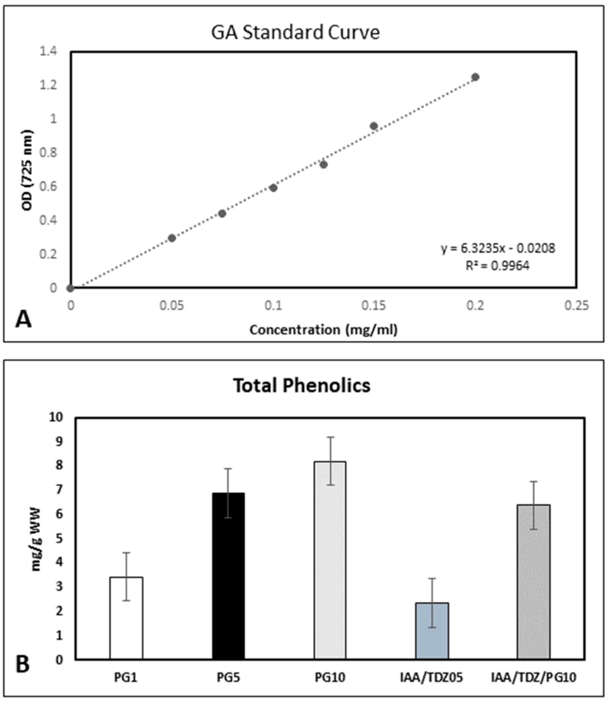

Figure S2. In Panel A, standard curve generated using Gallic Acid (GA) as monomeric phenolic. In panel B, total phenolics evaluation under PG and IAA/TDZ and IAA/TDZ+PG treatments. (WW: wet weight).

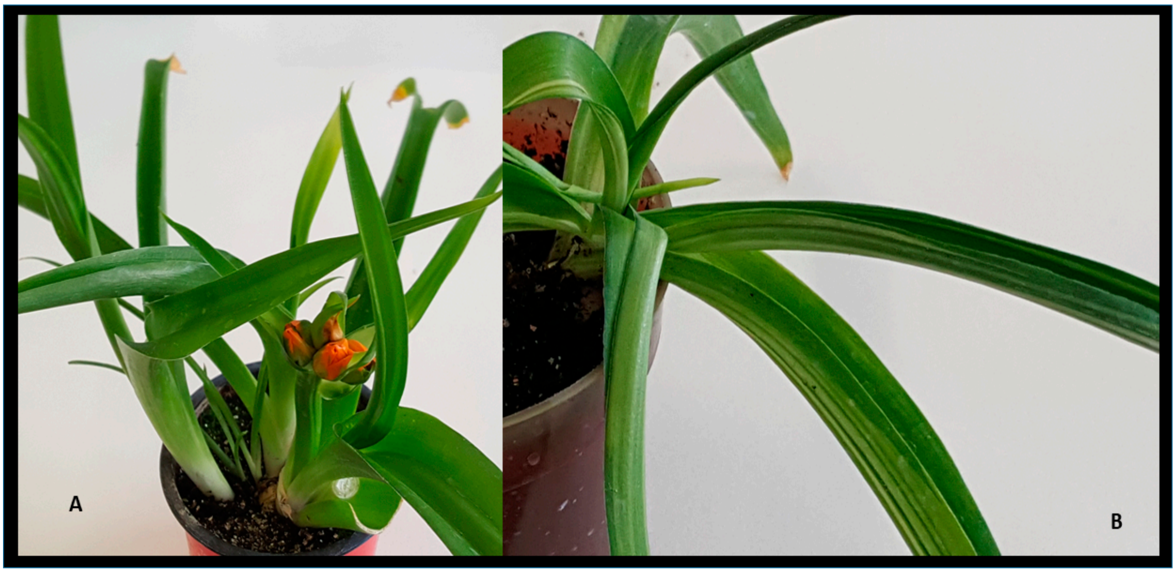

Figure S3. Morphological characteristic of the *O. dubium* leaf variegation variant. In B the typical morphology of a variegation variant as compared to a normal developed plant and leaf in A.

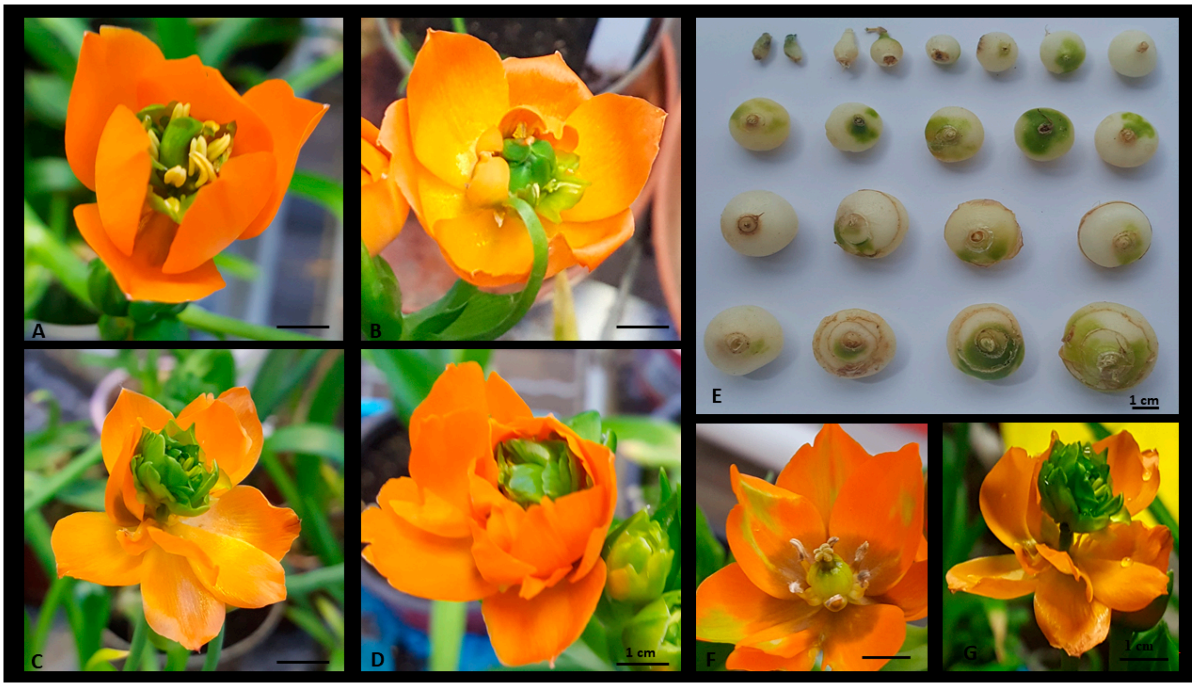

Figure S4. Plant Phenotyping of selected *Ornithogalum dubium* lines showing various morphological alteration of the flower development of *O. dubi*um. Increase in petals and stamens (in Panels B, C, D and G), green phenotype (in Panel F) and leafy pedunculated pistil (in panel G). In Panel E, normally developed bulbs of various sizes.

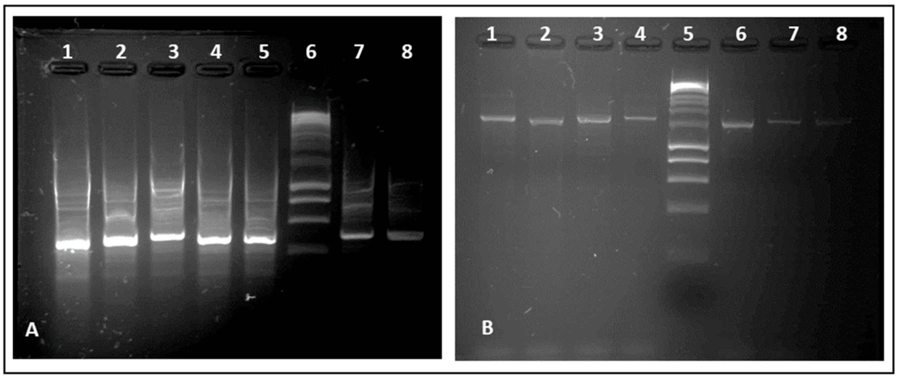

Figure S5. In A an example of a polymorphic primer (S24) applied to 7 variants. In B an example of a non-polymorphic primer (S25) applied to 7 variants. Lane 1: normal phenotype, 2: Variegated sample; 3: Micro-leaves sample; 4: Phloroglucinol induce, large sample; 5: Mixed coloured flowers; 6: 100 bp ladder and 7: green flower sample and 8: *flore pleno* sample
